# Post-Irradiation Study of the Alanine Dosimeter

**DOI:** 10.6028/jres.119.011

**Published:** 2014-07-14

**Authors:** Marc F. Desrosiers

**Affiliations:** National Institute of Standards and Technology, Gaithersburg, MD 20899

**Keywords:** alanine, dosimetry, electron paramagnetic resonance, gamma radiation, stability, time

## Abstract

Post-irradiation stability of high-dose dosimeters has traditionally been an important measurement influence quantity. Though the exceptional stability of the alanine dosimeter response with time has rendered this factor a non-issue for routine work, the archival quality of the alanine dosimeter has not been characterized. Here the alanine pellet dosimeter response is measured up to seven years post-irradiation for a range of absorbed doses. This long-term study is accompanied by an examination of the environmental influence quantities (e.g., ambient light) on the relatively short-term (3–4 month) stability of both pellet and film commercial dosimeters. Both dosimeter types demonstrated exceptional stability in the short term and proved to be relatively insensitive to common influence quantities. The long-term data revealed a complex dose-dependent response trend.

## 1. Introduction

An important influence quantity in ionizing radiation dosimetry is the time interval between irradiation and measurement of a dosimeter. This is a key consideration for dye-based radiochromic dosimeters that experience post-irradiation optical density changes with time [1]. The successor to the radiochromic system, alanine dosimetry, has demonstrated exceptional post-irradiation response stability that is key to postal-based transfer dosimetry services offered by calibration laboratories. The dosimetry system is based on irradiated crystalline alanine measured by electron paramagnetic resonance (EPR) spectrometry. Advancements over the past two decades have enabled the alanine-EPR system to become a world-class transfer reference system that is the centerpiece of high-dose dosimetry services for National Metrology Institutes (NMI) [2]. Moreover, the high quality of the dosimeter measurements, its relative low sensitivity to environmental influences and handling ease over dye-based systems have hastened the implementation of the alanine dosimetry system for routine use in industrial process dosimetry.

Though relatively stable with respect to the calibration service period, a change in the EPR response for irradiated alanine dosimeters is measurable with time [3–6]. For a post-irradiation time frame that includes hours, days or weeks depending on the application, the measurement changes are generally considered to remain within the measurement uncertainty (≈2 %). High-precision time-dependent studies of irradiated alanine pellet and film dosimeters revealed dose-dependent complexities within the measurement uncertainty from the early minutes to several days [3–6]. A year-long study that focused on alanine pellet relative humidity (RH) effects measured changes of several percent that increased with dose and the RH of the local environment [6]. Key to the accuracy of long-term temporal dosimeter studies is the use of an EPR internal reference for measurement reproducibility. The NIST reference material, synthetic ruby, is sensitive to the EPR measurement environment and configuration [7]. When alanine is measured in tandem with the ruby reference accurate long-term measurement comparisons are possible. Based on these features, alanine has been commonly regarded as a dosimeter that can be stored/archived and retrieved if required and reread at a later date, presumably years. Though universally accepted, this commonly stated attribute is derived from projections of (relatively) short-term data and anecdotal experiences. In practice, if an archived present-day commercial alanine dosimeter was selected for measurement several years post-irradiation, the response correction factor to be applied to the dosimeter measurement and its associated uncertainty are highly speculative.

A straightforward approach to assessing the alanine time dependence would be to irradiate a select group of alanine dosimeters and record their response over several years. Ideally, multi-year studies should employ an internal reference material such as synthetic ruby to compensate for the inevitable changes to a measurement system that may include minor changes to the sample holder configuration as well as more significant equipment repair and upgrades. Considering these possibilities, a study of this type is not practical since the EPR measurement system is very sensitive to the internal design and configuration of the microwave resonator. For success, the system configuration would be required to remain constant (and preferably undisturbed) for multiple years. Another consideration would be the selection of dosimeters for the multi-year study; they would need to be representative of the entire dosimeter batch/lot and formulation. The last and most obvious consideration is that the results of the study would require many years to complete.

An elegant solution to assessing the long-term dosimeter response profile arose from common practices for the alanine dosimetry system that supports the NIST transfer dosimetry services. The validity of the calibration curve for the NIST dosimetry system is assessed through the use of check standards [8]. The check standards are calibrated alanine dosimeters that are irradiated to each of the following doses: 25 Gy, 200 Gy, 1 kGy, 10 kGy, and 50 kGy. These check standards are measured prior to transfer dosimetry service measurements. They are also measured periodically to monitor the system performance during periods of service measurement gaps. These data are compiled into a control chart for tracking and system maintenance [8]. Check standards at these five dose levels were routinely stored as part of the system records. The check standards were used to maintain five different calibration ranges: 20 Gy to 100 Gy; 0.1 kGy to 1 kGy; 1 kGy to 10 kGy; 10 kGy to 70 kGy; and (less frequently) customized ranges above 70 kGy. Check standards for doses outside of the 20 Gy to 70 kGy range were generated and measured as needed and were not archived. Dosimetry services below 1 kGy were not routine in the early history of the NIST services and as such the check dose levels with the longest history (up to five years) are 1 kGy, 10 kGy and 50 kGy. This collection of check standards presented a unique opportunity to assess the archival potential of the alanine system because for the entire multi-year period the dosimeters were derived from a single manufactured batch and irradiated with gamma-ray sources in identical geometries that were calibrated and traceable to the NIST standard for absorbed dose [8]. The solution presented is to measure these archived check standards on the same day in a single session. Each group of dosimeters irradiated on a specific date would have since undergone their intrinsic changes with time and by combining all of these measurements at a specific point in time, a multi-year temporal study of the alanine system could be constructed within a few hours.

There are influence quantities that are not captured by this long-term temporal study. As these check standards were all stored identically, the study does not capture potential laboratory environmental influences such as temperature, relative humidity, and ambient light. Concerns for measurement temperature and relative humidity are effectively removed by using an EPR internal reference material such as ruby in a controlled analytical laboratory environment. No formal investigation of ambient light influence has ever been performed on currently available commercial dosimeters. However, several years ago a study of the effects of sunlight and fluorescent light on irradiated alanine was published that claimed these effects were significant and should be taken into account in alanine dosimetry [9]. This study claimed alanine EPR signal losses greater than 50 % for 290 h of light illumination of pure crystalline alanine powders, and EPR signal losses of about 2 % per hour of sunlight for self-described “commercial” alanine films. However, a contradictory report of ambient light insensitivity for a custom-manufactured alanine dosimeter has been published [10]. To date, the applicability of these findings to present-day commercial dosimeters and measurement practices in a metrology laboratory setting remains unknown. For insight into the alanine system as used in a modern metrological setting, a study was conducted that served both to assess the influence of environmental influence quantities on alanine pellet and film commercial dosimeters over the period of common use, the short-term (days/weeks) immediately following irradiation.

## 2. Experimental

Alanine pellet dosimeters were measured with a Bruker Biospin ECS106 EPR spectrometer. The EPR response is corrected for dosimeter mass and is normalized to an *in situ* ruby reference standard. The EPR measurement parameters for alanine pellet measurements below 1 kGy were: center field, 345.5 mT; microwave power, 0.25 mW; magnetic field sweep width, 2.0 mT; modulation amplitude, 1.43 mT; time constant, 2.6 s. The EPR measurement parameters for alanine pellet measurements above 1 kGy were: center field, 345.5 mT; microwave power, 0.50 mW; magnetic field sweep width, 1.0 mT; modulation amplitude, 0.285 mT; time constant, 1.3 s. Alanine film dosimeters were measured with a Bruker Biospin EMX EPR spectrometer. The EPR response is normalized to an *in situ* ruby reference standard. The EPR measurement parameters for alanine film measurements were: center field, 330.0 mT; microwave power, 0.25 mW; magnetic field sweep width, 50.0 mT; modulation amplitude, 0.90 mT; time constant, 0.08 s.

The alanine pellet dosimeters (Lot T030901) used for this study were distributed by Far West Technology (Goleta, CA). The alanine film dosimeters (Lot B339) were provided by Bruker Biospin USA (Billerica, MA). The coefficient of variation for alanine dosimeter measurements is 0.5 % for pellet dosimeters and 1.5 % for film dosimeters.

The irradiations for this study were performed using either of three Gammacell 220 ^60^Co irradiators (Nordion, Canada): serial number 207 with a dose rate of 6.6 kGy/h; serial number 232 with a dose rate of 1.3 kGy/h; or, serial number 45 with a dose rate of 0.40 kGy/h. The calibration scheme for determining the dose rate has recently been detailed [8]. Irradiation geometries have been published previously [11].

## 3. Results and Discussion

### 3.1 Alanine Pellet Long-Term Response

**Fig. 1:**
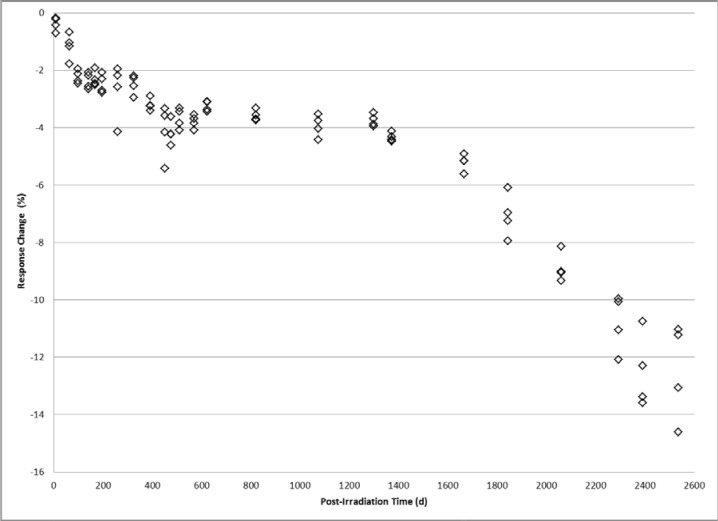
Alanine dosimeter measurement response displayed as the percent difference relative to the calibration response at 1.0 kGy versus the number of days post-irradiation.

For alanine system multi-dose comparisons an absorbed dose of 1 kGy is a preferred starting point. Relative to doses higher and lower, it is considered to have a strong EPR signal that is stable, within the linear portion of the broad dose range of the system, and unaffected by dose rate [8]. Figure 1 shows the relative response for 1 kGy calibrated alanine dosimeters measured from 8 days to 2,535 days after irradiation. The check dose standards used for the study are comprised of four alanine pellet dosimeters irradiated together. The response is relative to the regression-predicted measurement response at 1 kGy from the current system calibration curve. The time course for the dosimeter response is not a continuous loss of signal but rather an eventual loss of signal marked by periods of relative stability. For 1 kGy the first response plateau occurs between 97 days and 325 days with an approximate 2 % loss in signal. Another plateau occurs between 451 days and 1372 days at which a 4 % reduction in signal is held approximately constant. Measurements from 1,666 days to 2,535 days display a progressive loss in signal that also reveals an increase in the standard deviation of each group of four co-irradiated dosimeters. In approximate terms, at the end of a period of about seven years, the 1 kGy dosimeters experienced a cumulative loss in signal of 12 %.

**Fig. 2:**
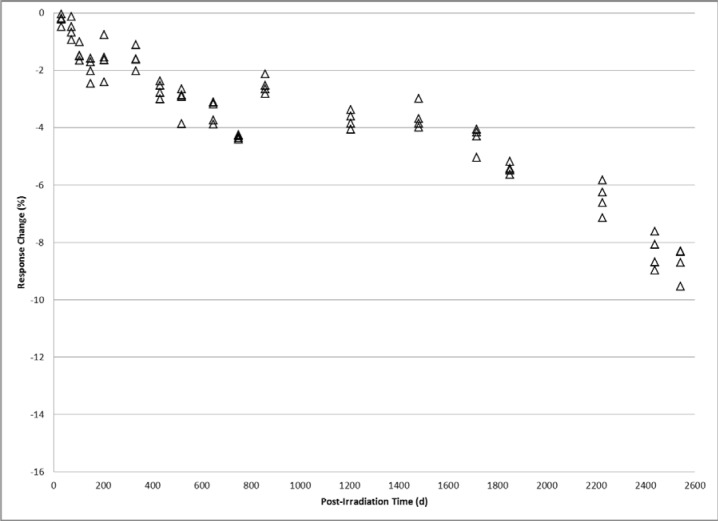
Alanine dosimeter measurement response displayed as the percent difference relative to the calibration response at 10 kGy versus the number of days post-irradiation.

**Fig. 3:**
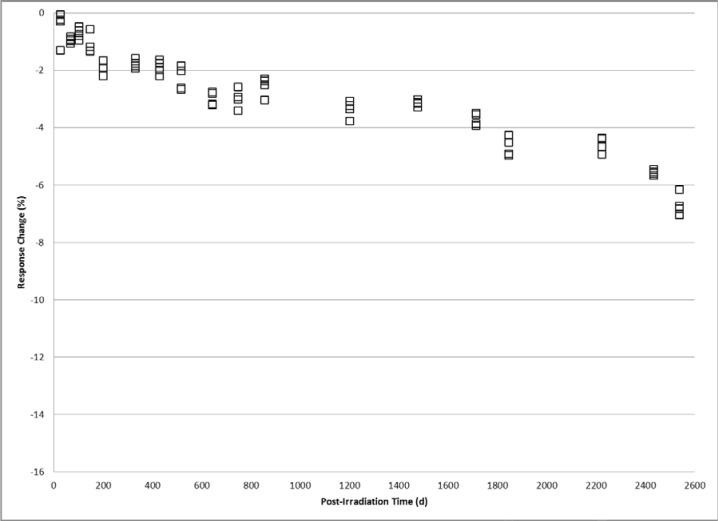
Alanine dosimeter measurement response displayed as the percent difference relative to the calibration response at 50 kGy versus the number of days post-irradiation.

Trends comparable to the 1 kGy study are observed in the time dependence of 10 kGy alanine dosimeters. The relative response of 10 kGy dosimeters measured from 30 days to 2,543 days is shown in Fig. 2. Plateaus in the signal fading pattern equivalent to 2 % and 4 % signal reduction levels are observed between 105 days and 333 days, and between 519 days and 1480 days, respectively. The cumulative loss in signal (≈9 %) after approximately seven years is not as large as that measured for 1 kGy. At 50 kGy the response trend with time resembles that of the 1 kGy and 10 kGy but the plateau region features are less resolved and the magnitude of the changes not as great. Figure 3 shows the post-irradiation response from 27 days to 2,539 days for 50 kGy alanine dosimeters. Plateaus in the signal fading pattern are visible but less defined at 2 % and 3 % reduction levels that occur between 201 days and 428 days, and 644 days and 1477 days, respectively. The cumulative loss in signal after about seven years was approximately 6.5 %.

**Fig. 4:**
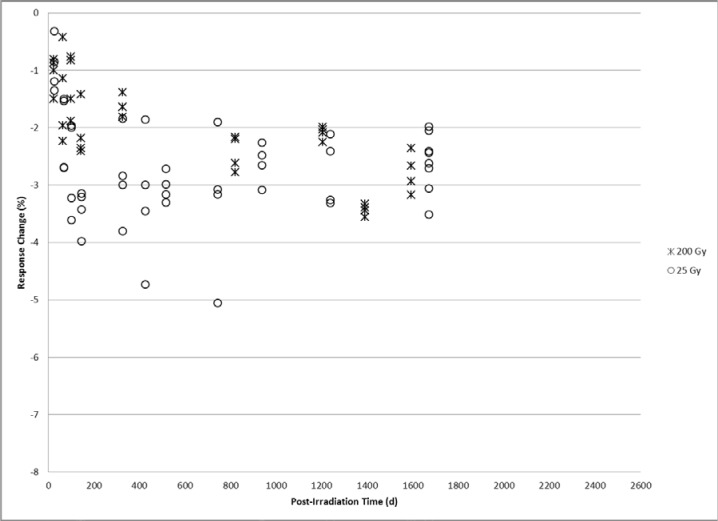
Alanine dosimeter measurement response displayed as the percent difference relative to the calibration response at 200 Gy (asterisk) and 25 Gy (circle) versus the number of days post-irradiation.

The post-irradiation alanine dosimeter response is relatively featureless for the two doses measured below 1 kGy. As shown in Fig. 4 for 25 Gy and 200 Gy measured from about 25 days to 1600 days the response drops to the 2 % to 3 % range at about 100 days and remains essentially constant. The relatively large signal scatter associated with the 25 Gy data is attributable to the relatively weak signal at that dose level.

### 3.2 Alanine Pellet Short-Term Response

Of particular practical interest to dosimetry is the stability of the alanine dosimeter in the immediate days and weeks following irradiation. Alanine is the transfer dosimeter of choice to establish traceability for end-users to national standards as well as for international comparisons between National Metrology Institutes. It is not uncommon for delays of one to three weeks to occur between irradiation and measurement. Though there is expert consensus that the modern commercial alanine dosimeter is stable during this post-irradiation period, the bulk of the published supporting data either pre-dates commercially available dosimeters [3,6] or employs unconventional methods [4]. The multi-year studies shown here for commercially available dosimeters sparked a detailed investigation for the time period immediately following irradiation. Such a study presents an opportunity to also examine the potential effects of the laboratory environment on post-irradiation stability. Again, expert consensus considers the alanine dosimeter response to be robust and insensitive to influence quantities common to the ambient conditions of an analytical laboratory [12]. In opposition stands a study that claims strong light effects on the irradiated alanine dosimeter [9]. If the light effect claims are applicable to current commercial systems, the ambient light of the laboratory could conceivably influence the dosimeter response. While it lies outside the scope of the present study to conduct a formal investigation of light effects, a time-dependent study was devised that could capture an effect by comparing irradiated dosimeters stored protected in a laboratory cabinet versus irradiated dosimeters resting exposed to the ambient conditions of the laboratory. The plan is based on the expectation that good laboratory practices are consistently followed in a modern laboratory. While a mechanistic photochemical study of the crystal-bound alanine-derived free radical is a worthy academic pursuit, this study is directly relevant to the metrological system as used in practice.

**Fig. 5:**
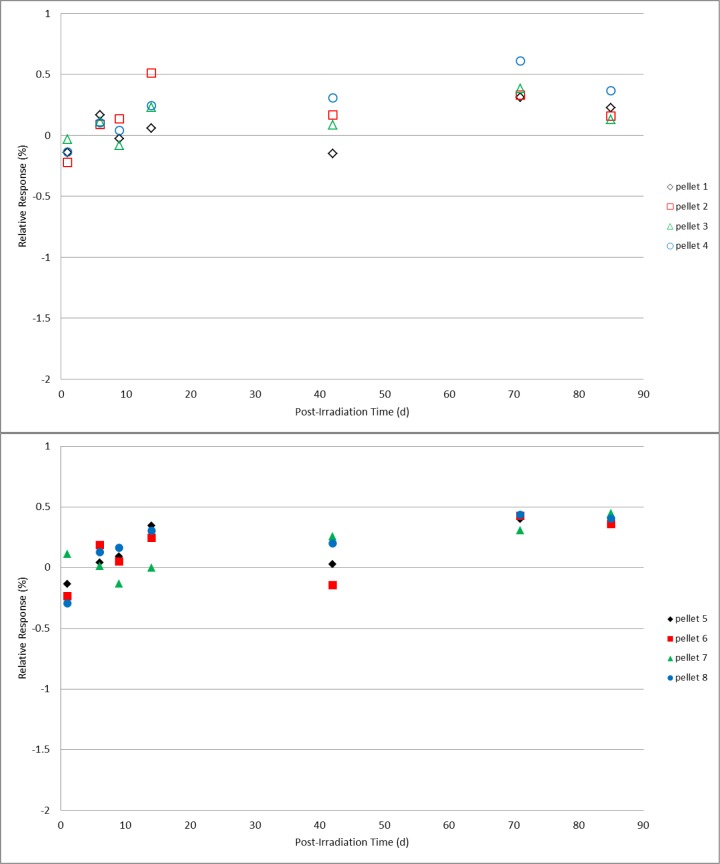
Relative response for 0.2 kGy individual alanine dosimeters versus the number of days post-irradiation. The top graph (open symbols) represents pellets stored unprotected and exposed to the laboratory environment; the dosimeter measurements in the bottom graph (solid symbols) are for pellets stored in a protected environment.

**Fig. 6:**
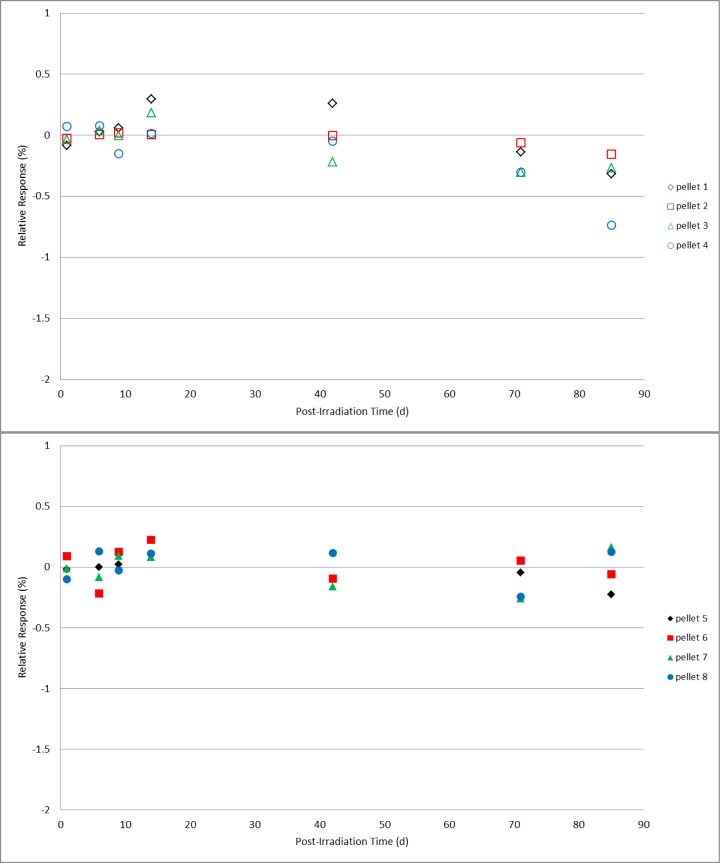
Relative response for 1.0 kGy individual alanine dosimeters versus the number of days post-irradiation. The top graph (open symbols) represents pellets stored unprotected and exposed to the laboratory environment; the dosimeter measurements in the bottom graph (solid symbols) are for pellets stored in a protected environment.

**Fig. 7:**
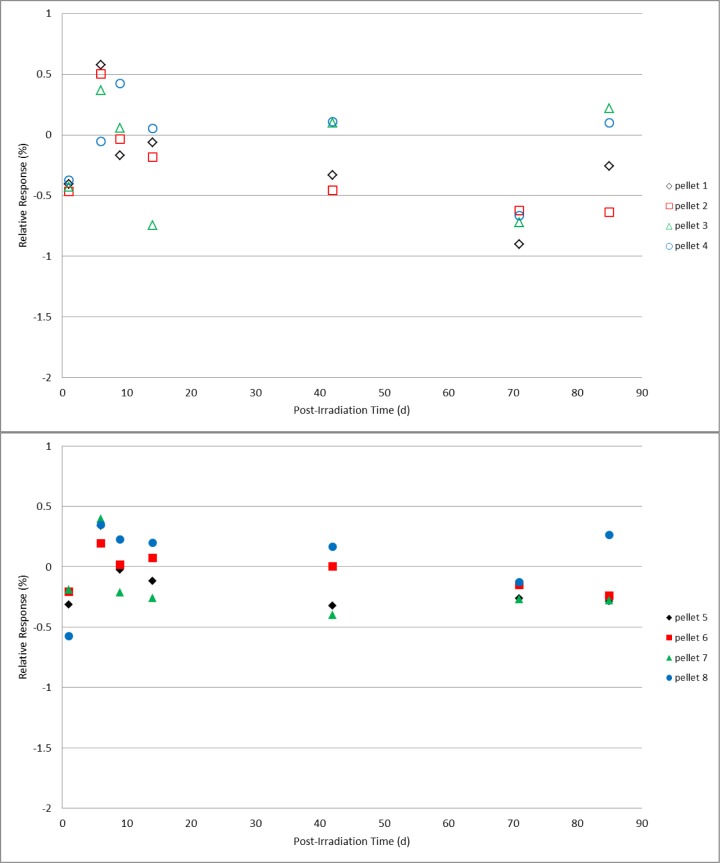
Relative response for 10 kGy individual alanine dosimeters versus the number of days post-irradiation. The top graph (open symbols) represents pellets stored unprotected and exposed to the laboratory environment; the dosimeter measurements in the bottom graph (solid symbols) are for pellets stored in a protected environment.

**Fig. 8:**
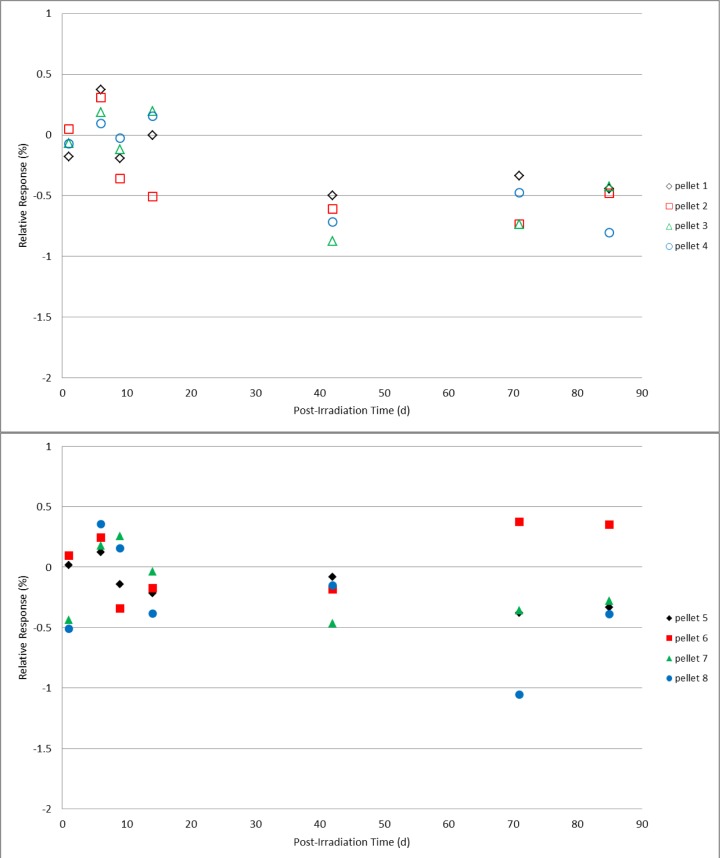
Relative response for 40 kGy individual alanine dosimeters versus the number of days post-irradiation. The top graph (open symbols) represents pellets stored unprotected and exposed to the laboratory environment; the dosimeter measurements in the bottom graph (solid symbols) are for pellets stored in a protected environment.

Two sets of four alanine pellet dosimeters were irradiated under identical conditions to doses of 25 Gy, 200 Gy, 1 kGy, 10 kGy, and 40 kGy. Post-irradiation, one set was spread into a single layer on a plastic tray that was covered but not sealed and placed in a closed laboratory cabinet that also was not sealed from the environment but could be considered protected from direct light and air flow. The second set of dosimeters rested in a single layer uncovered in a plastic tray that was placed in a location that would ensure exposure to the most significant variations in laboratory conditions; under room fluorescent lighting near a large external window and near the room’s heating/cooling ventilation system. Measurements were made on days 1, 6, 9, 14, 42, 71, and 85 following irradiation. During the measurement period (several hours) for each day no special precautions were taken to shield the protected group dosimeters from the ambient light and environment. A mean value for the response specific to each group of four dosimeters was determined from multiple measurements and served as the reference value for that group of dosimeters. All subsequent measurements were calculated relative to that mean value. For reference, the expected relative standard deviation for the measured response of a group of co-located irradiated alanine dosimeters is ≈0.5 %. The results for 200 Gy and 1 kGy (Figs. 5 and 6), with the exception of a couple of single measurements, remained within their expected values for both unprotected and protected dosimeters over the 85 day duration. Though a small deviation is apparent in the 10 kGy results for the unprotected dosimeters at 71 days, the results at 85 days were equivalent (Fig. 7). At the highest dose studied, 40 kGy, a small but consistently measurable difference is evident between the unprotected and protected dosimeters after 42 days (Fig. 8). For the lowest dose, 25 Gy, data plots of the type shown in Figs. 5–8 are not appropriate due to the weaker signal and significant influence of the background signal of the sample cavity. To analyze the 25 Gy data, a mean value for each dosimeter group was determined and the ratio of the unprotected set mean to the protected set mean was computed. The ratios exhibited no trend with time and averaged 1.00 ±0.6 % over the 85 day duration. Due to a required spectrometer maintenance event, the study (for all doses) was terminated at 85 days.

### 3.3 Alanine Film Dosimeters

For comparison to the alanine pellet dosimeter influence quantities study, two sets of four alanine film dosimeters were irradiated under identical conditions to doses of 10 kGy, 20 kGy, and 30 kGy. Post-irradiation, one set was grouped into a glass test tube that was open-topped, stood upright in a rack and placed in a closed laboratory cabinet that was not sealed from the environment but could be considered protected from direct light and air flow. The second set of dosimeters rested in a single layer (alanine side up) uncovered in a plastic tray that was placed in a location that would ensure exposure to the most significant variations in laboratory conditions; under room fluorescent lighting near a large external window that was in close proximity to the room’s ventilation system. To gather baseline data on the post-irradiation stability of alanine films across the full range of the dosimeter system an additional set of alanine films were irradiated under identical conditions to doses of 0.6 Gy, 0.8 Gy, 1 kGy, 2 kGy, 4 kGy, 4 kGy, 7 kGy, 50 kGy, 80 kGy, and 90 kGy. Post-irradiation, these dosimeters were placed in the protected environment of the closed laboratory cabinet as described above.

**Fig. 9:**
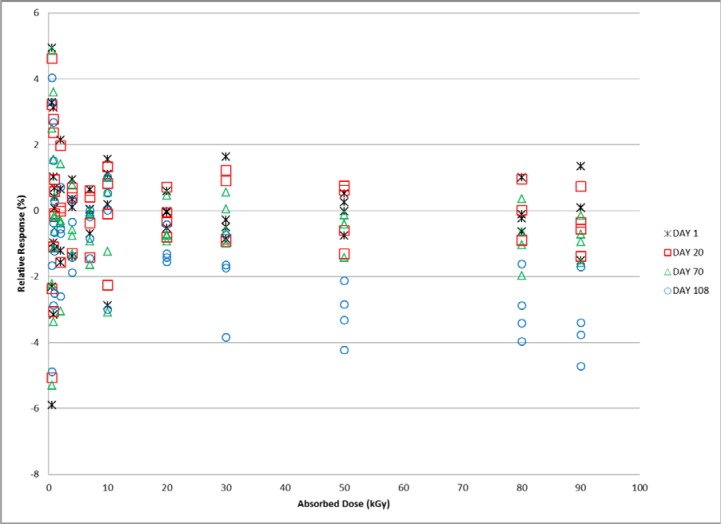
Relative response for alanine film dosimeters irradiated from 0.6 kGy to 90 kGy and measured on days 1, 20, 70, and 108 post-irradiation.

**Fig. 10:**
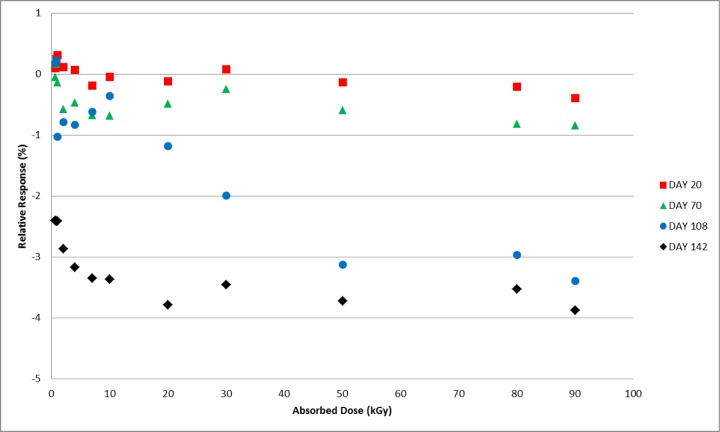
Mean of the relative response for four alanine film dosimeters irradiated from 0.6 kGy to 90 kGy and measured on days 20, 70, 108, and 142 post-irradiation.

Measurements were made on days 1, 20, 70, 108, and 142 following irradiation for the protected set of dosimeters irradiated to the full range of doses (0.6 kGy to 90 kGy). A mean value for the response specific to each group of four dosimeters was determined from the day 1 measurements and served as the reference value for that group of dosimeters. All subsequent measurements were calculated relative to that mean value. For reference, the expected variation for the measured response of a group of co-irradiated alanine film dosimeters is ≈1.5 %. Through day 70 the alanine response for all doses remained within the expected range of values (Fig. 9). Measurements made on day 108 revealed a dose dependent response variation. As shown in Fig. 9, the response for doses up to 10 kGy are equivalent to that measured on previous days. However, a small deviation is observable at 20 kGy and the deviation gets progressively larger with increasing dose. For clarity the data from Fig. 9 was converted from individual film measurements to the mean of four dosimeters measured together at each dose and the 142 day data were added for comparison in Fig. 10. A large and significant decrease in the relative response is apparent for all dose levels measured on the 142^nd^ day.

**Fig. 11:**
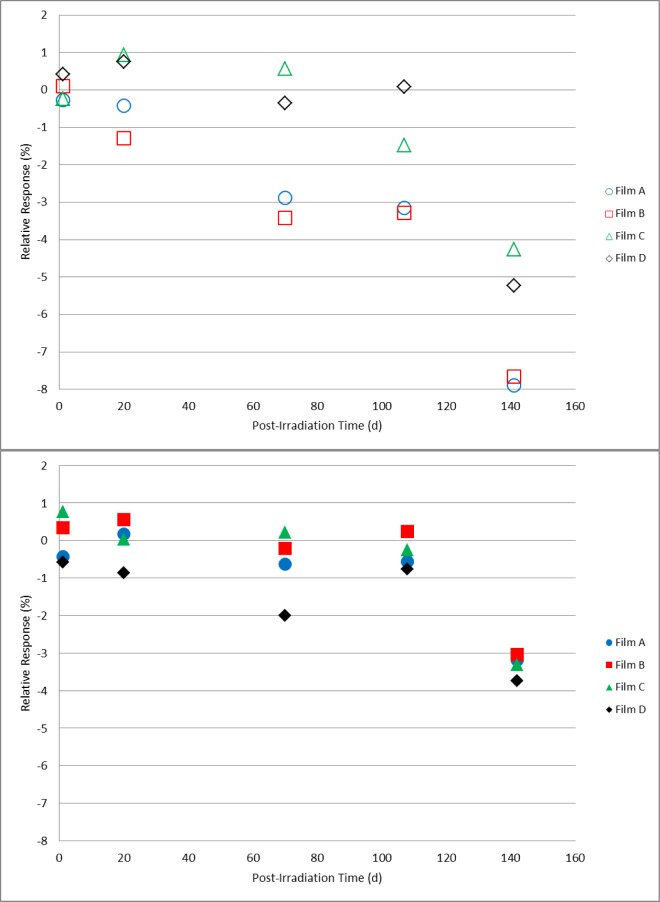
Relative response for 10 kGy alanine film dosimeters measured on days 20, 70, 108, and 142 post-irradiation. The top graph (open symbols) represents films stored unprotected and exposed to the laboratory environment; the dosimeter measurements in the bottom graph (solid symbols) are for films stored in a protected environment.

**Fig. 12:**
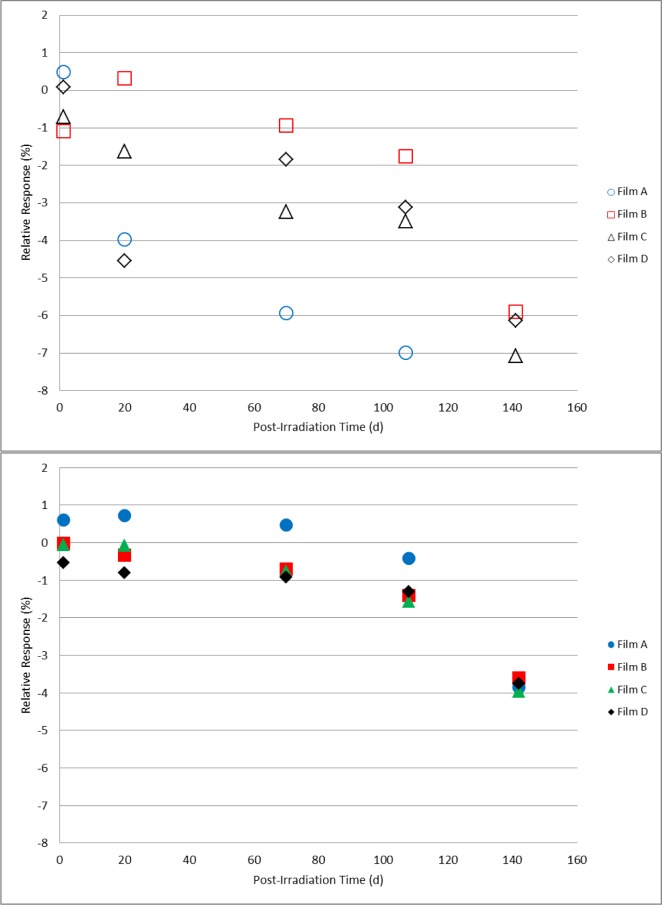
Relative response for 20 kGy alanine film dosimeters measured on days 20, 70, 108, and 142 post-irradiation. The top graph (open symbols) represents films stored unprotected and exposed to the laboratory environment; the dosimeter measurements in the bottom graph (solid symbols) are for films stored in a protected environment.

**Fig. 13:**
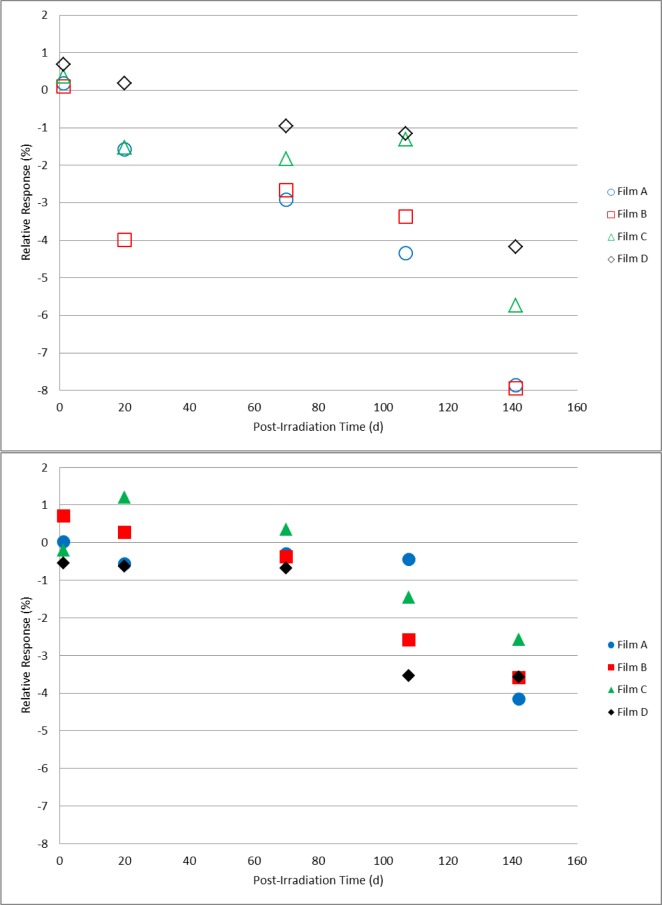
Relative response for 30 kGy alanine film dosimeters measured on days 20, 70, 108, and 142 post-irradiation. The top graph (open symbols) represents films stored unprotected and exposed to the laboratory environment; the dosimeter measurements in the bottom graph (solid symbols) are for films stored in a protected environment.

**Fig. 14:**
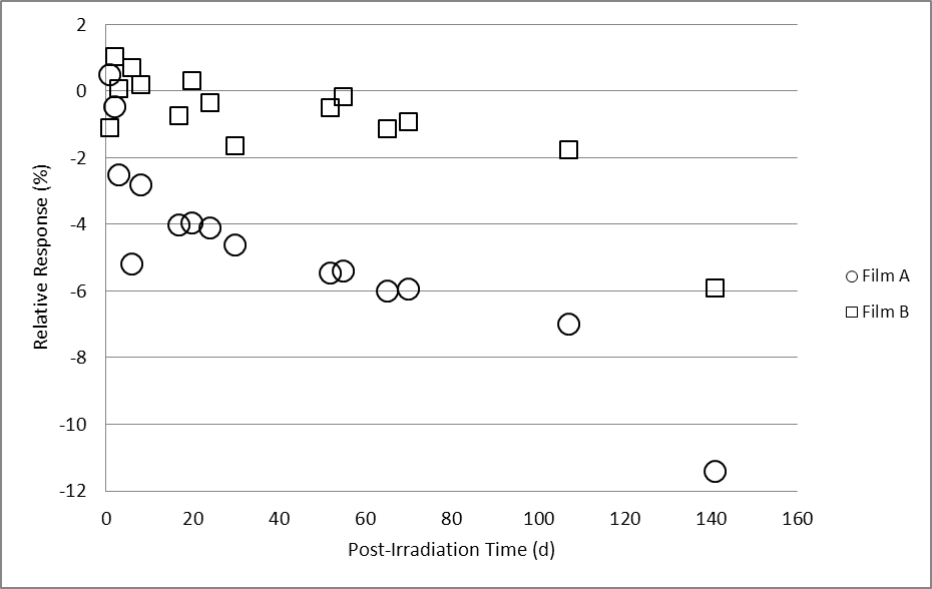
Relative response for two irradiated alanine film dosimeters measured on multiple days post-irradiation. The films were co-irradiated to 20 kGy and stored adjacent to each other unprotected and exposed to the laboratory environment.

For the paired sets of dosimeters irradiated to 10 kGy, 20 kGy, and 30 kGy and stored either in a protected or unprotected environment as described above, comparative measurements were made on days 1, 20, 70, 108, and 142 following irradiation. These data are shown in Figs. 11–13. For the dosimeters stored in the protected environment (Figs. 11b, 12b, 13b) the trends are consistent with that shown in Fig. 10. The measurements for all three doses were equivalent up to 70 days with 10 kGy being stable for 108 days, 20 kGy slightly decreased at 108 days and 30 kGy significantly decreased on day 108. On day 142 the response significantly decreased for all three doses. However, for the dosimeters that were unprotected and exposed to the laboratory environment significant changes were evident. The data (Fig. 11a) revealed a distinct divergence of the dosimeter response for two of the films relative to the other two films of the same dose and storage condition. Two of the four 10 kGy dosimeters in the unprotected group had a time course similar to the protected group while the other two experienced a significant loss of signal. For most of the 20 kGy dosimeters in the unprotected group the decrease in response was evident at 20 days and continued to decrease with time (Fig. 12a). However, a single film dosimeter from the 20 kGy unprotected group was an exception to the trend. One dosimeter displayed an early significant loss in signal that remained consistently distinct from the 20 kGy unprotected dosimeter group for the duration of the measurements. The response for 30 kGy dosimeters in the unprotected group progressively decreased with time for all films in a similar manner (Fig. 13a). Additional measurements were made for the films in the unprotected group (only). The expanded data set shown in Fig. 14 for two films co-irradiated and stored together provide a detailed example of how the temporal response can vary between film dosimeters irradiated and stored identically.

## 4. Conclusions

The findings here of complex response profiles for archived irradiated alanine dosimeters are both unexpected and valuable. Before these data, an attempt to accurately measure a stored alanine dosimeter may assume a long-term time dependence based on a projection of relatively short-term response trends. Response adjustments based on assumptions of a uniform decay profile would conceivably introduce errors on the order of several percent depending on the elapsed post-irradiation time. The data presented in Figs. 1–4 offers for a more accurate response adjustment and uncertainty estimate. Dose-dependent differences are apparent from these data, but considering the large dose range (25 Gy to 50 kGy) they are remarkably similar for the multi-year post-irradiation period. The source of the multi-featured temporal response is unknown. However, the free radical chemistry of irradiated crystalline alanine is known to involve multiple radical species [13]. The spectrum, and thus the measured response, represents the sum of a distribution of radical concentrations at a specific point in time. It is reasonable to assume that over time these radical species, each with a specific decay profile, could spectrally combine to yield the observed complex response trends.

Check standards that are created to assess the quality of the alanine dosimetry system are typically used for one to two months (or as needed). Several years of experience using the alanine system for NIST transfer dosimetry services has demonstrated that, with few isolated exceptions, this period of stability is reproducible and that no extraordinary storage conditions are necessary to maintain this stability. As the majority of post-irradiation alanine studies were performed in the early development stages [3,6], very little has been published on this topic for modern commercial alanine dosimeters [4,5]. The only direct study of the effect of light on the irradiated alanine dosimeter primarily examined non-commercial dosimeters treated under extraordinary conditions [14]. At the current precision level for the alanine system (±1 %), it was expected that, if the light sensitivity claims of 2 % per hour were applicable to ambient light, these effects would be measureable. Comparisons were made between alanine pellet dosimeters placed in protected and unprotected laboratory locations. The unprotected location was considered a worst case for the NIST environment: an open tray of dosimeters in a single layer near the room’s air handling vents in direct view of a large external window and directly under the room fluorescent lighting. Intentionally, extraordinary precautions were not taken for the protected dosimeters; they were in a covered container placed in a closed cabinet neither of which was environmentally sealed. For alanine pellets irradiated from 25 Gy to 10 kGy (Figs. 5–7), prolonged exposure (85 days) to the laboratory environment clearly had no effect while at 40 kGy (Fig. 8) only a borderline effect that remained within the acceptable measurement limits (±1 %). On average the protected dosimeters produced more consistent measurements over time. Though a protected storage environment is advisable, extraordinary measures (e.g., light-shielded humidity-controlled storage) are not required.

A parallel protected/unprotected storage study with alanine film dosimeters gave varied results that suggested a possible film-to-film difference in environmental sensitivity (Figs. 11–13). The protected group gave consistent results throughout the study. Surprisingly, measurement trends for dosimeters from the unprotected group diverged from each other even within an isodose group. Figure 14 represents a more detailed example of this observation. The data is plotted for two dosimeters irradiated together and stored side-by-side. While one dosimeter demonstrated response stability (1 % loss up to day 70) the other decayed rapidly (≈1 %/d within the first 3–5 days). These observations may be due to the high surface-to-volume ratio of the film versus pellet as well as suggest that the film dosimeter may contain minor manufacturing variations that enable the surface-bound irradiated alanine crystals to be susceptible to environmental influences. It bears repeating that the protected group displayed no environmental effects. Nonetheless, good practice would dictate that irradiated alanine films be closely monitored for the best results.

The post-irradiation stability of commercial alanine films has been examined previously though not extensively [5]. Figures 9 and 10 demonstrate that these dosimeters offer excellent stability over the full dose range of use for the system for up to 70 days. For doses 10 kGy and below the period of stability can be extended to 108 days. These dosimeters were stored in a protected location (*vide supra*) and appropriate care would be expected to achieve these results.

There was no evidence that the significant photochemical effects measured previously by other researchers apply to modern commercial alanine dosimeters. Though an effect was observed for *specific* film dosimeters, these data do not distinguish between any of the potential influences alone or combined, they include: ambient light sources, temperature, relative humidity, or exposure to air flow. The alanine pellet dosimeter showed no such effects; however the surface-to-volume ratio for pellets differs from the films as well as the method of manufacture and polymer binder material. For the irradiated dosimeter to be significantly sensitive to visible light the radical species would need to be capable of absorbing those wavelengths, i.e., have absorption bands at wavelengths in the visible spectrum. Whereas, anecdotally, it is common to observe yellow toning on the normally colorless alanine dosimeter at doses greater than 10 kGy, it is unknown whether the color arises from the measured paramagnetic alanine radical, an accumulation of radiation-induced degradation products, or both. Diffuse reflection spectroscopy studies cite an absorption maximum for irradiated crystalline alanine in the near UV at ≈350 nm [10]. Those studies claim anecdotally that “light had little influence on the radiation induced radical”, but that “long illumination with light of … 354 nm” caused photobleaching of the alanine radical [10]. The alanine radical has previously been shown to be photosensitive to UV wavelengths [15]. The results presented here are consistent with an insensitivity of alanine dosimeters to ambient light and provide no support for the prior claims of hypersensitivity to visible light.

The conditions in the Ciesielski study that produced photoeffects in irradiated alanine were extreme relative to that commonly experienced in an analytical laboratory [14]. Their studies employed either direct sunlight or multiple light sources mounted in close proximity (3 cm) to the dosimeters. In addition, both light sources contributed to a rise in temperature (up to 48 °C for sunlight) and the authors considered temperature effects to be negligible (due to separate thermal-only studies). However, the possibility of enhanced reactivity for the alanine radical due to photoexcitation at elevated temperatures should not be discounted. Another potential flaw in the experimental design is that the UV and IR components of the light source were not filtered. It is considered good practice to remove these components in visible wavelength photochemical studies [16]. While the IR component contributes to the thermal effects, the UV component could influence the photochemistry. In fact, a case for the effect caused by UV excitation is supported by the authors observation that, “the sensitivity to light was decreasing with increase in dose” [14]. As mentioned above, alanine dosimeters turn increasingly yellow with gamma-irradiation dose. This color is indicative of absorption bands in the UV and near-UV portion of the spectrum. If UV excitation of the alanine radical is responsible for the observed effects, a reduction in the effect with dose is consistent with an attenuation of the incident light by an increasing accumulation of yellow radiation-induced degradation products. The data presented here, coupled with the interpretation of the unusual observations of Ciesielski [8,14] as flaws in their experimental design and analysis, support the conclusion that the environmental conditions required to initiate photoeffects in irradiated alanine lie outside that commonly found in a modern laboratory environment.

Recommendations for good measurement practices that arise from this work are that irradiated alanine dosimeters may be stored in a protected environment without the need for extraordinary environmental controls if the EPR spectrometer employs an internal reference material. Irradiated alanine check standards would be expected to be stable for at least two months under these conditions. However, as found here in particular for film dosimeters, the possibility exists for individual dosimeters to exhibit sensitivity to environmental influence quantities and the use of multiple dosimeters at each dose level accompanied by consistent monitoring are recommended for check standard applications.
